# The perceptions and experiences of women with a body mass index ≥ 30 kg m^2^ who breastfeed: A meta‐synthesis

**DOI:** 10.1111/mcn.12813

**Published:** 2019-04-26

**Authors:** Stephanie Lyons, Sinead Currie, Sarah Peters, Tina Lavender, Debbie M. Smith

**Affiliations:** ^1^ Manchester Centre for Health Psychology, Division of Psychology and Mental Health, School of Health Sciences, Faculty of Biology, Medicine, and Health The University of Manchester Manchester UK; ^2^ Psychology, Faculty of Natural Sciences University of Stirling Stirling UK; ^3^ Centre for Global Women's Health, Division of Nursing, Midwifery and Social Work, Faculty of Biology, Medicine and Health The University of Manchester Manchester UK; ^4^ School of Social and Health Sciences Leeds Trinity University Horsforth UK

**Keywords:** BMI, breastfeeding, experiences, obesity, perceptions, systematic review

## Abstract

Breastfeeding has copious health benefits for both mother and child, but rates of initiation and maintenance among women with a body mass index (BMI) ≥ 30 kg m^2^ are low. Few interventions aiming to increase these rates have been successful, suggesting that breastfeeding behaviour in this group is not fully understood. Therefore, this review aimed to systematically identify and synthesise the qualitative literature that explored the perceptions and experiences of women with a BMI ≥ 30 kg m^2^ who breastfed. The search identified five eligible papers, and a meta‐ethnographic approach was taken to synthesise the findings. One theme was identified: “weight amplifies breastfeeding difficulties,” revealing that women with a BMI ≥ 30 kg m^2^ experience common breastfeeding difficulties to a greater degree. In particular, women with a BMI ≥ 30 kg m^2^ struggle with the impact of medical intervention, doubt their ability to breastfeed, and need additional support. These findings can inform understanding of breastfeeding models, future research directions, intervention development, and antenatal and post‐natal care for women with a BMI ≥ 30 kg m^2^.

**Key messages**

- Women with a body mass index (BMI) ≥ 30 kg m^2^ are less likely to breastfeed than women with a BMI ≤ 30 kg m^2^.
- Women with a BMI ≥ 30 kg m^2^ experience common breastfeeding difficulties to a greater degree due to their weight.
- Women with a BMI ≥ 30 kg m^2^ struggle with the impact of medical intervention, doubt their ability to breastfeed, and need additional support.
- It is recommended that health professionals empower women with a BMI ≥ 30 kg m^2^ to breastfeed despite medical intervention, address women's beliefs about their ability to produce nutritionally adequate milk, and fully explain and discuss the purpose of breastfeeding support.

## 1 INTRODUCTION

Globally, rates of obesity (i.e., body mass index [BMI] ≥ 30 kg m^2^) are increasing (Ng et al., 2014). This results in increased rates of cardiovascular disease, type 2 diabetes, and some cancers and places huge economic stress on employers, governments, and health care systems (Hojjat & Hojjat, 2017; Lehnert, Sonntag, Konnopka, Riedel‐Heller, & Konig, 2013), in addition to impacting negatively upon individual health and well‐being and life expectancy (Alston & Okrent, 2017). Therefore, it is vital that obesity rates are reduced.

Breastfeeding can help to reduce obesity (Stuebe, 2009) as it reduces the risk of childhood obesity for the infant (Yan, Liu, Zhu, Huang, & Wang, 2014) and can help mothers to lose weight gained in pregnancy (Vinter et al., 2014). Breastfeeding is particularly beneficial for women with a BMI ≥ 30 kg m^2^, as they are more likely than normal weight women (i.e., BMI: 18–24.99 kg m^2^) to gain excessive weight in pregnancy (Restall et al., 2014), and their infants are more likely to become obese in childhood (Yu et al., 2013). However, women with a BMI ≥ 30 kg m^2^ are less likely than normal weight women to initiate and maintain breastfeeding for at least 6 months (Jarlenski et al., 2014).

Despite the clear need for breastfeeding interventions directed at women with a BMI ≥ 30 kg m^2^, few have been published (Carlsen et al., 2013; Chapman et al., 2013; Rasmussen, Dieterich, Zelek, Altabet, & Kjolhede, 2011). Moreover, in contrast to interventions targeting all women (Sinha et al., 2015), most were unsuccessful at increasing breastfeeding duration. The one intervention that reported an increase in breastfeeding duration was not representative of all women with a BMI ≥30 kg m^2^ (Carlsen et al., 2013). Together, this suggests that more understanding of the facilitators and barriers to breastfeeding behaviour in women with a BMI ≥30 kg m^2^ is needed in order to better inform targeted and effective interventions.

Psychological factors can facilitate and prevent breastfeeding behaviour (de Jager, Broadbent, Fuller‐Tyszkiewicz, & Skouteris, 2014). A recent systematic review that investigated the role of psychological factors in breastfeeding behaviour in women with a BMI ≥ 30 kg m^2^ (Lyons, Currie, Peters, Lavender, & Smith, 2018) found that planning to breastfeed, believing in the nutritional adequacy and sufficiency of breast milk, believing that important others approve of breastfeeding, knowing others who have breastfed, and having positive body image were associated with better breastfeeding outcomes. However, this review included only quantitative studies, and therefore, the conclusions are limited to those factors, which had been investigated in studies using psychometric measures, and do not include factors emerging from women's lived experiences. Therefore, the current review aims to systematically synthesise the qualitative literature that explores the perceptions and experiences of women with a BMI ≥30 kg m^2^ who breastfed.

## 2 METHODS

### 2.1 Design

A systematic approach was taken to identifying and reviewing all qualitative research that has explored the perceptions and experiences of women with a BMI ≥30 kg m^2^ who report engaging in breastfeeding behaviours. The synthesis was informed by Noblit and Hare's (1988) method of meta‐ethnography. This method was appropriate for the analysis as it can be used to synthesise studies that have employed different qualitative methods (Campbell et al., 2011; Elmir, Schmied, Wilkes, & Jackson, 2010). Included studies were identified by conducting a systematic search.

### 2.2 Search strategy

Following a scoping exercise to develop the research question and suitable search terms, an electronic systematic search was conducted on the Cumulative Index to Nursing and Allied Health Literature, PsycINFO, and PubMed databases in August, 2017. The OpenGrey, MedNar, and Trove databases were used to search grey literature. Multifield search builders were used to combine keywords in accordance with the SPIDER framework (see Table 1; Cooke, Smith, & Booth, 2012). Authors and journals of included studies were also hand‐searched to minimise the possibility of missing relevant articles.

**Table 1:** Keywords for each search term

SPIDER reference	Search terms
Sample	Obes* OR body mass index OR bmi OR body mass index 30 OR bmi 30 OR overweight
Phenomenon of interest	Breastfee* OR breast fe* OR lactat* or infant feeding
Design	Questionnaire* OR survey* OR interview* OR focus group* OR case stud* OR observ*
Evaluation	View* OR experience* OR opinion* OR attitude* OR perce* OR belie* OR feel* OR know* OR understand*
Research type	Qualitative OR mixed method*

*Note*. * represents truncation. Combined [S AND P of I] AND [(D or E) AND R].

### 2.3 Eligibility criteria

This synthesis included studies that investigated the perceptions and experiences of women with a BMI ≥30 kg m^2^ who reported breastfeeding behaviours. Therefore, included studies focused on women's perceptions and/or experiences of breastfeeding who had a BMI ≥30 kg m^2^ at the start of their pregnancy. All studies had to include qualitative methods of both data collection and analysis.

Studies that focused on health professionals' perceptions of these women's experiences were included; in this case, only relevant themes were extracted (i.e., those themes that depicted factors that influenced the women's breastfeeding behaviours). Studies that included subsamples were included if their views were reported separately from those of women with a BMI ≥30 kg m^2^. Included studies were restricted to those written in English due to a lack of funding to translate non‐English papers, with no date restrictions set.

### 2.4 Study selection and quality appraisal

**Figure 1:**
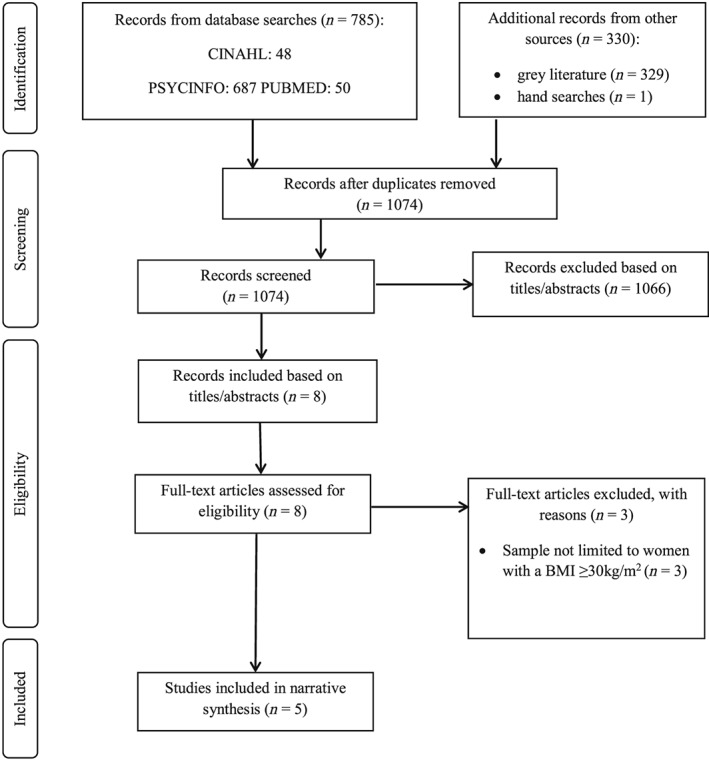
PRISMA flow diagram of study selection

Search results were entered into EndNote ×7, and duplicates were removed. Titles and abstracts were screened by one researcher (S. L.), and those that did not meet the eligibility criteria were excluded. At this stage, an inter‐rater reliability assessment was conducted, with a second researcher (D. M. S.) also reviewing 10% of title and abstracts (Kitchenham, 2004). The inclusion/exclusion decision of the second researcher was compared against the firsts, generating a Cohen's kappa statistic. The level of agreement was “very good,” κ = 0.823 (95% CI [0.584, 1.00]), *P* < .0005. Full texts were then retrieved for remaining studies, and two researchers assessed each study against the eligibility criteria. At this stage, the level of agreement was “good,” κ = 0.75 (95% CI [0.306, 1.00]), *P* = .028. The process of study selection is illustrated in a PRISMA flow diagram (Figure 1).

To allow comparison between included studies, the Critical Appraisal Skills Programme Qualitative Research Checklist (2017) was used. This involves assessing the aims, design, recruitment strategy, data collection, researcher bias, ethical issues, data analysis, findings, and implications of each study and awarding “yes” if the item is met, “no” if it is not, or “unclear” if the necessary information is not available. As the quality appraisal did not form part of the eligibility criteria, a scoring system was not applied.

### 2.5 Data extraction and synthesis

Data extraction and synthesis followed the meta‐ethnography process described by Noblit and Hare (1988; Table 2). This first involved scoping the literature and identifying an appropriate area of interest and research question. Eligibility criteria were created and applied to determine which studies were included in the synthesis. Included studies were read and re‐read by two researchers, with key phrases and themes highlighted and combined into lists. Two researchers completing this step reduced the risk of excluding relevant studies (Kitchenham, 2004). These lists also included information on the study aims, sample, methods, and form of analysis. The list for each study was then compared and contrasted with the list for each other study by three researchers, and the relationships between studies were determined. This resulted in the translation of studies into one another, and a line of argument was developed. Three researchers completing this step prevent bias and improve conceptual development (Morse, 2015). The results of the synthesis are expressed in this paper.

**Table 2:** Descriptions of the phases of synthesis

Phase	Description
Getting started	Research team scoped available literature and identified an appropriate area of interest that qualitative methodology can inform.
Deciding what is relevant to the initial interest	Research team decided and defined the scope of the synthesis and set eligibility criteria.
Reading the studies	Two researchers read and re‐read the studies, highlighting important phrases and themes.
Determining how the studies are related	Two researchers created lists of the key phrases and themes for each study. Draw initial assumptions about how included studies relate to one another.
Translating the studies into one another	Three researchers compared and contrasted the lists for each study and identified concepts that were encompassed across studies.
Synthesising translations	Three researchers ensured the translation encompassed key concepts and themes of included studies and compared translation to alternative interpretations. A line of argument was developed from the final translation.
Expressing the synthesis	Research team considered the potential audience of the synthesis and expressed results in an appropriate format.

## 3 RESULTS

The systematic search identified 1,115 papers, with 1,074 remaining after duplicates were removed (Figure 1). The titles and abstracts were then reviewed, resulting in the exclusion of 1,066. Eight papers were reviewed at full text, with five included in the synthesis. For four of the five studies, women with a BMI ≥ 30 kg m^2^ were interviewed about their perceptions and experiences of breastfeeding; for the remaining study (Massov, 2015), health professionals were interviewed on their experiences of providing care for BMI ≥ 30 kg m^2^ who breastfeed. Included studies employed either content or thematic analysis. All studies were high quality. Study characteristics are summarised in Table 3, and individual quality item scorings for each study can be found in Table S1.

**Table 3:** Study characteristics of included studies

Study reference	Aim	Country	Sample and recruitment	Method of data collection	Method of analysis
Garner et al. (2014) (1)	• To understand how health professionals across the continuum of care perceive breastfeeding among obese women. • To understand how health professionals experience providing care to obese women to identify potential barriers and ways to improve breastfeeding‐related care.	USA	• Thirty‐four health professionals in a range of roles (e.g., lactation consultants, nurse midwife, and nurses), and with experience ranging from 5 to 30 years. • Recruited participants by sending emails through listservs to health professionals in obstetrics, midwifery, family medicine, and paediatric practices.	Face‐to‐face, semistructured interviews	Content analysis
Garner et al. (2016) (2)	• To understand obese women's experiences and perceptions longitudinally, with a normal‐weight comparison group, to identify key experiences and barriers that are unique to or more common among obese women.	USA	• Thirteen pregnant obese (BMI ≥ 30 kg m^2^) and nine normal‐weight (BMI: 18.5–24.9 kg m^2^) women who intended to breastfeed. • Recruited through flyers and brochures placed in a variety of medical settings.	Face‐to‐face, semistructured interviews	Content analysis
Keely et al. (2015) (3)	• To explore the factors that influence breastfeeding practices in obese women who either had stopped breastfeeding or were no longer exclusively breastfeeding, despite intending to exclusively breastfeed for at least 16 weeks.	Scotland	• Twenty‐eight women with a BMI >30 kg m^2^ at the start of pregnancy, who breastfed at the first feed, and intended to breastfeed for at least 16 weeks, but were no longer exclusively breastfeeding at 6–8 weeks. • Recruited participants on the post‐natal ward.	Face‐to‐face, semistructured interviews	Thematic analysis
Lyons et al. (2019) (4)	• To explore the experiences of women with a BMI ≥ 30 kg m^2^ who have breastfed.	England	• Eighteen women with a BMI ≥ 30 kg m^2^ at the start of pregnancy who had breastfed and/or were currently breastfeeding at the time of the interview. • Recruited through online adverts posted on breastfeeding support groups and related forums.	Telephone, semistructured interviews	Thematic analysis
Massov (2015) (5)	• To explore the breastfeeding experiences and perspectives of clinically overweight and obese women. • To gain an understanding of what influenced their infant feeding decisions.	New Zealand	• Six women with a BMI > 30 kg m^2^ at the start of their pregnancy who had initiated breastfeeding, but were no longer exclusively breastfeeding at 4–6 weeks. • Recruited through Lead Maternity Carer, who identified eligible women from their hospital records.	Face‐to‐face, semistructured interviews	Thematic analysis

Abbreviation: BMI, body mass index.

The synthesis identified one theme: Weight amplifies breastfeeding difficulties. The way in which the included papers mapped on to the theme and subthemes is illustrated in Table 4. Quotes from included papers are provided to support the synthesis findings.

**Table 4:** Illustration of how individual paper findings map on to synthesis theme and subthemes

Theme: Weight amplifies breastfeeding difficulties					
Subthemes	Garner et al. (2014)	Garner et al. (2016)	Keely et al. (2015)	Lyons et al. (2019)	Massov (2015)
Psychological reactions to medical interventions	• Women with a BMI > 30 kg m^2^ have reduced stamina and mobility due to weight and caesarean sections. • Women with a BMI > 30 kg m^2^ have low motivation because medical interventions make breastfeeding more difficult.	• Women with a BMI > 30 kg m^2^ often have long, difficult labours, which may result in caesarean section and reduced mobility and infection. • Women with a BMI > 30 kg m^2^ had sick babies and were separated from their infants.	• Caesarean sections delay skin‐to‐skin and lead to separation from infants. • Medical interventions lead to longer hospital stays, where women with a BMI > 30 kg m^2^ feel uncomfortable to initiate breastfeeding due to the lack privacy.	• Many women with a BMI > 30 kg m^2^ experience medical intervention, which removes their sense of control over their pregnancy and breastfeeding.	—
Perception of body's ability to breastfeed	• Women with a BMI > 30 kg m^2^'s body shape and size negatively impact on breastfeeding, and they fear smothering their infant. • Women with a BMI > 30 kg m^2^ worry about their body's ability to breastfeed and are self‐conscious because they struggle to breastfeed discreetly. • Positioning requires more trial and error.	• Women with a BMI > 30 kg m^2^ lacked confidence in their ability to breastfeed, so planned to breastfeed for shorter durations. • Women with a BMI > 30 kg m^2^ have difficulty with positioning and latching, and their perception of their body size and shape leads to fears of suffocating their infant. • Women with a BMI > 30 kg m^2^ struggle to find affordable nursing bras in the right sizes.	• Women with a BMI > 30 kg m^2^ lacked belief in their ability to breastfeed discreetly in public and did not want to breastfeed in front of family and friends in their home, which led to the cessation of exclusive breastfeeding. • Having a bigger body and breasts caused difficulty with latching and positioning and led women to fear suffocating their infant. • Women with a BMI > 30 kg m^2^ introduced formula milk early due to beliefs that they were not producing enough breast milk.	• Women with a BMI > 30 kg m^2^ face barriers related to their body size and shape, which reinforce the perceived lack of control over breastfeeding. • Women with a BMI > 30 kg m^2^ worry about their ability to produce nutritious milk.	• Women with a BMI > 30 kg m^2^ found breastfeeding difficult and felt unprepared. • Having difficulty latching because of larger breasts and bodies led women with a BMI > 30 kg m^2^ to lack belief in their ability to breastfeed and to do so discreetly and to worry about suffocating their infant. • Women with a BMI > 30 kg m^2^ introduced formula milk due to beliefs that they did not produce enough breast milk.
Additional need for support	• Women with a BMI > 30 kg m^2^ have less access to social support compared with other women. • Women with a BMI > 30 kg m^2^ need more support and education about breastfeeding in pregnancy.	•Women with a BMI > 30 kg m^2^ needed more latching support over a longer period of time but had less access to social support.	• Partner support was viewed as important when making infant feeding decisions. • Few women with a BMI > 30 kg m^2^ sought professional support because they misunderstood its purpose, but those that did described it as a positive experience. • Women with a BMI > 30 kg m^2^ wanted support at home and valued support from friends who breastfed.	• Seeking support helped women with a BMI > 30 kg m^2^ to overcome medical, social, and practical barriers. • Women with a BMI > 30 kg m^2^ wanted support beyond the initial latching stage to normalise and support maintenance.	• Women with a BMI > 30 kg m^2^ sometimes found professional help distressing, as they were not comfortable with health professionals touching their bodies and infant. • Women with a BMI > 30 kg m^2^ felt uncomfortable asking for professional help in hospital as they felt staff were too busy.

Abbreviation: BMI, body mass index.

### 3.1 Weight amplifies breastfeeding difficulties

This theme describes how difficulties that many women experience while initiating and maintaining breastfeeding are exaggerated in women with a BMI ≥ 30 kg m^2^ because of their weight. For example, although medical intervention while giving birth can lead to breastfeeding difficulties for all women (Rowe‐Murray & Fisher, 2002), women with BMI ≥ 30 kg m^2^ are likely to report suffering complications and becoming separated from their infants (Garner, McKenzie, Devine, Thornburg, & Rasmussen, 2016; Keely, Lawton, Swanson, & Denison, 2015; Lyons, Currie, & Smith, 2019) and, therefore, perceive a lack of control over their infant feeding behaviours, which negatively impacts breastfeeding. Furthermore, although many women may doubt their ability to breastfeed (Avery, Zimmermann, Underwood & Magnus, 2009), women with BMI ≥ 30 kg m^2^ encounter more barriers, which reduce their confidence in their body, and negatively impact their perception of their ability to breastfeed (Garner et al., 2016; Garner, Ratcliff, Devine, Thornburg, & Rasmussen, 2014; Keely et al., 2015; Lyons et al., 2019; Massov, 2015). Lastly, although gaining the right breastfeeding support is important for all women (Backstrom, Hertfelt Wahn, & Ekstrom, 2010), women with BMI ≥ 30 kg m^2^ appear to need additional help throughout their breastfeeding journeys (Garner et al., 2014; Garner et al., 2016; Keely et al., 2015; Lyons et al., 2019; Massov, 2015). Therefore, the theme had three subthemes: (a) psychological reactions to medical intervention, (b) perception of body's ability to breastfeed, and (c) additional need for support.

#### 3.1.1 Psychological reactions to medical intervention

This subtheme describes how psychological reactions to medical intervention during pregnancy and birth can have a negative impact on breastfeeding initiation and maintenance. Women with a BMI ≥30 kg m^2^ are often labelled as “high risk” in pregnancy (Lyons et al., 2019) due to their higher risk of experiencing long labours, which result in caesarean section, subsequent infections, longer hospital stays, reduced mobility, delayed skin‐to‐skin contact, and even periods of separation from their infant (Garner et al., 2014; Garner et al., 2016; Keely et al., 2015). During this, women often feel disappointed about their breastfeeding experience and unmotivated to continue, as there are already numerous barriers making breastfeeding difficult (Garner et al., 2014; Lyons et al., 2019). In particular, women with a BMI ≥ 30 kg m^2^ may not feel comfortable to breastfeed in the busy hospital environment, where privacy can be difficult to achieve (Keely et al., 2015). Due to this, women feel that their choice of infant feeding method is out of their control, which has a negative impact on breastfeeding initiation and maintenance (Lyons et al., 2019). As the likelihood of experiencing medical intervention during pregnancy and birth is higher among women with a BMI ≥ 30 kg m^2^ (Weiss et al., 2004), these difficulties are amplified within this group. Actively seeking professional and social support can help women to overcome these barriers and breastfeed (Lyons et al., 2019).

I was just shaking all over, like complete shaking so I was like, “I don't feel like I can hold him” (Eve; 3)

And then I got really bummed because she was only able to stay on me for a couple of minutes. The nursery came and got her because her blood sugar levels were low (Allison; 1)

#### 3.1.2 Perception of body's ability to breastfeed

This subtheme describes how lacking belief in your ability to breastfeed and, in particular, your body's ability to produce adequate and sufficient milk can have a negative impact on breastfeeding initiation and maintenance. For this reason, women either did not plan to breastfeed or did so only for short periods of time to avoid disappointment, which negatively impacted initiation and maintenance (Garner et al., 2016). Furthermore, difficulty latching and positioning due to larger breasts and bodies led women to feel unprepared and fear smothering their infant and reinforced their lack of belief in their ability to breastfeed (Garner et al., 2014; Garner et al., 2016; Keely et al., 2015; Lyons et al., 2019; Massov, 2015). Women also lacked belief in their ability to breastfeed discreetly, as their baby would cover less of their larger breasts and body, which led them to give their infant formula milk when family members or friends were visiting their homes, or they were out in public (Garner et al., 2014; Keely et al., 2015; Lyons et al., 2019; Massov, 2015). Because of their size, women also worried about the nutritional quality and sufficiency of their breast milk; women believed their diet quality may be poorer than women with lower BMIs and that this would translate directly to their breast milk quality, meaning many women introduced formula milk early due to concerns that their infant was still hungry after breastfeeding (Keely et al., 2015; Lyons et al., 2019; Massov, 2015). Other barriers, such as poor availability of nursing bras and tops in larger sizes, also reinforced women's lack of belief in their ability to breastfeed (Garner et al., 2016; Lyons et al., 2019). Seeking professional support to assist with latching and positioning, social support to normalise breastfeeding for women with a BMI ≥ 30 kg m^2^, and information to correct beliefs about their ability to produce nutritious and sufficient milk helps women to overcome these barriers and initiate and maintain breastfeeding (Lyons et al., 2019).

She's probably over 300 pounds and tried to breastfeed but this poor little baby, you know, you try and nurse a little tiny baby and their nose is right next to their mouth, like truly was smothered by this woman's breasts. And I think the effort that it would go into for her to breastfeed was just too much. (MD; 2)

Nine hours or something like that trying to feed him. He was just crying and I remember … I could hear his belly rumble. He was starving and he obviously wasn't getting anything, I was sitting and I was in tears and I said to [my husband] “what am I going to do?” and he says “he needs to eat, so you need to give him formula” so I did (Nancy; 3)

#### 3.1.3 Additional need for support

This subtheme describes how women with a BMI ≥ 30 kg m^2^ need more support, not only while initiating but also while maintaining breastfeeding. This is because women with a BMI ≥ 30 kg m^2^ often have more difficulty latching and positioning, meaning they need more professional support to help them successfully initiate (Garner et al., 2014; Garner et al., 2016; Lyons et al., 2019). Women also valued social support from family members and friends, and particularly other women with a BMI ≥30 kg m^2^ who were breastfeeding, as they could share their experiences and ask for advice and tips, which improved their confidence (Keely et al., 2015; Lyons et al., 2019). This, in particular, helped women to normalise breastfeeding beyond 6 months, as many women had little social support available to them and had not met women who had breastfeed infants beyond this point (Garner et al., 2014; Garner et al., 2016; Lyons et al., 2019). However, there were barriers that prevented support seeking. For example, many women did not seek professional support because they felt they may be irritating busy health professionals, and they were uncomfortable breastfeeding in front of others, or with health professionals touching their bodies and infant (Massov, 2015). Furthermore, many women did not attend breastfeeding clinics due to a misconception of their purpose; some women believed that groups were only for women who had trouble with their latch, others believed that groups were only for women who were successfully breastfeeding, and some believed that the groups were for social support, but felt uncomfortable attending alone (Keely et al., 2015).

She was like you need to go to the local breastfeeding support group you know you'll get support from women like you and it'll, it'll help your confidence, and it was the best thing I did, definitely (Chloe; 4)

I just remember the midwife coming in and almost angry that I was upset because I was having trouble doing it and “I'll show you how to express” but then just pretty much grabbed my breast without asking if that was OK. And being really, really rough and aggressive with her and I kind of … it's not on … She just ruined the whole stay in hospital, like then it made me apprehensive about any other midwife that walked in the room and what were they going to do to me? (Jess; 5)

## 4 DISCUSSION

This review aimed to explore the perceptions and experiences of women with a BMI ≥ 30 kg m^2^ who breastfeed. Five studies were included. The findings highlight that these women experience similar difficulties to other women but that these are more problematic for women with a BMI ≥ 30 kg m^2^ because of their weight. These findings can help health care professionals and interventions to target their support, increase breastfeeding initiation and duration, and ultimately reduce obesity and obesity‐related diseases in women with a BMI ≥30 kg m^2^ and their children.

All women face the possibility of experiencing medical intervention in pregnancy and labour (Bhattacharya, Campbell, Liston, & Bhattacharya, 2007). However, women with a BMI ≥ 30 kg m^2^ are more likely to suffer complications during their pregnancy and labour, such gestational diabetes and hypertension, pre‐eclampsia, preterm delivery, stillbirth, and post‐partum haemorrhage, resulting in higher rates of hospital stays, inductions, and elective and emergency caesarean sections (Athukorala, Rumbold, Wilson, & Crowther, 2010; Bhattacharya et al., 2007). The findings highlight that these complications and interventions are barriers to breastfeeding and can leave women lacking the motivation and perceived control over their infant feeding behaviours to overcome them, which can partially explain why breastfeeding rates among women with a BMI ≥ 30 kg m^2^ are particularly low. Therefore, it is recommended that health care professionals are aware of the impact of these experiences on women with a BMI ≥30 kg m^2^ and provide assistance and encouragement where necessary to allow women to feel in control of their infant feeding behaviours and breastfeed. However, it is interesting that one study (Massov, 2015) did not identify the impact of medical intervention, despite recruiting their sample in a country that has low rates of “normal” spontaneous vaginal births (i.e., 33% New Zealand vs. 59% in England, 56% in Scotland, and 58% in the United States; Ministry of Health, 2015; NHS Digital, 2017; NHS Scotland, 2016; Declerq, Sakala, Corry, Applebaum, & Herrlich, 2013). One explanation for this may be that most women in New Zealand choose a midwife to deliver all of their care (i.e., throughout pregnancy and birth), where particular emphasis is placed on creating a partnership for decision making (Page, 2001; Ministry of Health, 2015), meaning these women may feel more in control of any medical intervention they receive, reducing its impact on breastfeeding. However, this approach to care is also taken in the United Kingdom (Royal College of Midwives, 2014). As breastfeeding rates in New Zealand are already high (i.e., 86% of women are breastfeeding to some extent at 6 weeks post‐partum; Plunket, 2019) compared with the United Kingdom (i.e., only 46% of women breastfeeding to some extent at 6 weeks post‐partum; Public Health England, 2019) and the United States (i.e., only 79% of women initiate breastfeeding; National Center for Chronic Disease Prevention and Health Promotion, 2014), it may also be that medical intervention has a similar influence on these women's sense of motivation and control, but their environment better supports breastfeeding behaviour and reduces other barriers. Therefore, further research is needed to fully determine the influence of medical intervention on breastfeeding in women with a BMI ≥ 30 kg m^2^. This could be investigated quantitatively, by tracking the interventions women receive and measuring their sense of motivation to and control over breastfeeding and, qualitatively, by specifically exploring the impact of medical intervention on breastfeeding in semistructured interviews.

Women often doubt their ability to breastfeed (Moore & Coty, 2006). However, the findings of this study suggest that women with a BMI ≥ 30 kg m^2^, in particular, are concerned about their ability to produce adequate and nutritious milk, which prevents them from planning to breastfeed. Furthermore, this study highlights that women with a BMI ≥ 30 kg m^2^ lack confidence in their ability to breastfeed in social situations. Quantitative studies have shown that lacking belief in the quality and sufficiency of breast milk and confidence to breastfeed publically is associated with negative breastfeeding outcomes (Hauck, Fenwick, Dhaliwal, & Butt, 2011; Stuebe & Bonuck, 2011), meaning the study findings can offer some explanation for the low breastfeeding rates in this group. Therefore, it is recommended that these beliefs are addressed in pregnancy, to encourage women to feel confident in their body's ability to breastfeed and to make infant feeding plans. In particular, addressing beliefs about initial infant feeding frequency may reduce women's worry that the breast milk they produce is insufficient. Once breastfeeding, it is recommended that women are signposted to breastfeeding support groups, in order to build their confidence to breastfeed in social situations.

Professional and social support can be beneficial for all breastfeeding women (Hannula, Kaunonen, & Tarkka, 2008; Ingram, Rosser, & Jackson, 2005). However, women with a BMI ≥ 30 kg m^2^ have additional needs for professional support during initiation; their body shape and size means that they often require more trial and error when latching and positioning. Women with a BMI ≥ 30 kg m^2^ also often know few others who have breastfed, so have greater needs for social support. Despite their additional needs, the findings of this study highlight that women with a BMI ≥ 30 kg m^2^ feel uncomfortable asking for and receiving support and often misunderstand its purpose. This may explain why women participating in breastfeeding support interventions did not take up the maximum number of contacts they were offered (Carlsen et al., 2013; Chapman et al., 2013). Therefore, future interventions providing breastfeeding support may benefit from discussing its purpose and addressing women's views and concerns about accessing appointments. It is also recommended that during women's care, health care professionals discuss different support options, in order to decide what form may be suitable and acceptable for them.

Only five studies met the criteria and were included in the synthesis, which may limit the strength of the conclusions drawn. In particular, it is important to note that the studies included were all conducted in high‐income, English‐speaking countries, and therefore, the findings may not be applicable to other settings. However, this fits with the form of analysis, as meta‐ethnography aims to synthesis findings from closely related studies exploring specific phenomena, rather than make generalisations across fields (Britten et al., 2002; Campbell et al., 2003).

This study had several strengths. In particular, relevant studies were searched for systematically and assessed against a predefined inclusion criteria, minimising the risk of missing material and researcher bias during study selection (Moher, Liberati, Tetzlaff & Altman, 2010; Kitchenham, 2004). Furthermore, inter‐rater reliability checks were conducted, and included studies were appraised for their quality using a valid tool, increasing the strength of the conclusions drawn (Kitchenham, 2004). Finally, included studies reported the experiences of both women with a BMI ≥ 30 kg m^2^ who ceased breastfeeding early and those who continued to breastfeed for as long as they wished, increasing the applicability of the findings.

Several implications are generated from the findings. First, as few studies were eligible for inclusion in this synthesis, more qualitative research of these women's breastfeeding experiences is needed, with particular focus placed on the influence of care. For example, longitudinal qualitative studies conducted throughout women's pregnancies and breastfeeding experiences may highlight aspects of medical intervention and communication, which could be adapted to reduce the negative impact on breastfeeding behaviour. These studies may also highlight how health care professionals can best reassure women with a BMI ≥ 30 kg m^2^ that they are capable of producing nutritionally adequate and sufficient milk and that they can overcome barriers due to their body size or shape with professional and social support. The influence of medical intervention could also be investigated quantitatively, by measuring both the instances and types of medical intervention women experience and their motivation to and perceived control over breastfeeding. In order to increase breastfeeding behaviours among this group, intervention development is also necessary; future interventions should consider these findings, particularly those that offer breastfeeding support. Finally, it is recommended that health care professionals also consider these findings during their practice, by taking extra care to empower women with a BMI ≥ 30 kg m^2^ to breastfeed despite whether they have experienced medical intervention, address women's beliefs about their ability to breastfeed and produce nutritionally adequate milk in both pregnancy and post‐partum, and to fully explain and discuss the purpose of breastfeeding support, including different available options that are acceptable to each individual.

## 5 CONCLUSION

In conclusion, this study explored the perceptions and experiences of women with a BMI ≥ 30 kg m^2^ who breastfeed. It was found that experiencing medical intervention reduced women's sense of motivation and control over breastfeeding behaviours, the women doubted their ability to breastfeed, and often misunderstood the purpose of breastfeeding support, or felt uncomfortable to ask for help. These findings can inform understanding of breastfeeding models, future research directions, intervention development, and antenatal and post‐natal care.

## CONFLICTS OF INTEREST

The authors declare that they have no conflicts of interest.

## CONTRIBUTIONS

All researchers contributed to the design of the study. SL, DS, and SC completed the analysis. The article was drafted by SL. All authors critically revised the article and gave approval for the final version to be published.

## Supporting information

Table S1.
Quality appraisal results for included studies
