# Supplementary material for: The perceptions and experiences of women with a body mass index ≥ 30 kg m2 who breastfeed: A meta‐synthesis
Source: Matern Child Nutr. 2019 Apr 26;15(3):e12813. doi: 10.1111/mcn.12813 (PMC6618145; doi:10.1111/mcn.12813)
Supplement: Supplementary file 1 — Table S1. Quality appraisal results for included studies [file MCN-15-e12813-s001.docx]

**Supplementary Table 1.**

|  | Garner et al. (2014) | Garner et al. (2016) | Keely et al. (2015) | Lyons et al. (2019) | Massov (2015) |
| --- | --- | --- | --- | --- | --- |
| Was there a clear statement of the aims of the research? | Yes | Yes | Yes | Yes | Yes |
| Is a qualitative methodology appropriate? | Yes | Yes | Yes | Yes | Yes |
| Was the research design appropriate to address the aims of the research? | Yes | Yes | Yes | Yes | Yes |
| Was the recruitment strategy appropriate to the aims of the research? | Yes | Yes | Yes | Yes | Yes |
| Was the data collected in a way that addressed the research issue? | Yes | Yes | Yes | Yes | Yes |
| Has the relationship between researcher and participants been adequately considered? | Unclear | Unclear | Unclear | Unclear | Unclear |
| Have ethical issues been taken into consideration? | Yes | Yes | Yes | Yes | Yes |
| Was the data analysis sufficiently rigorous? | Unclear | Unclear | Unclear | Yes | Unclear |
| Is there a clear statement of findings? | Yes | Yes | Yes | Yes | Yes |
| Is the research valuable? | Yes | Yes | Yes | Yes | Yes |

**Supplementary Table 1.** Quality appraisal results for included studies.
